# Double-Serine Fluoroquinolone Resistance Mutations Advance Major International Clones and Lineages of Various Multi-Drug Resistant Bacteria

**DOI:** 10.3389/fmicb.2017.02261

**Published:** 2017-11-16

**Authors:** Miklos Fuzi, Dora Szabo, Rita Csercsik

**Affiliations:** Institute of Medical Microbiology, Semmelweis University, Budapest, Hungary

**Keywords:** double-serine, QRDR, International clones, multiresistant pathogens, promotion of dissemination

## Abstract

The major international sequence types/lineages of methicillin-resistant *Staphylococcus aureus* (MRSA), extended-spectrum β-lactamase (ESBL)-producing *Klebsiella pneumoniae* and ESBL-producing *E. coli* were demonstrated to have been advanced by favorable fitness balance associated with high-level resistance to fluoroquinolones. The paper shows that favorable fitness in the major STs/lineages of these pathogens was principally attained by the capacity of evolving mutations in the fluoroquinolone-binding serine residues of both the DNA gyrase and topoisomerase IV enzymes. The available information on fitness balance incurred by individual and various combinations of mutations in the enzymes is reviewed in multiple species. Moreover, strong circumstantial evidence is presented that major STs/lineages of other multi-drug resistant bacteria, primarily vancomycin-resistant *Enterococcus faecium* (VRE), emerged by a similar mechanism. The reason(s) why the major ST/lineage strains of various pathogens proved more adept at evolving favorable mutations than most isolates of the same species remains to be elucidated.

## Introduction

It is well-established that major international clones and lineages of various multi-drug resistant hospital-associated pathogens emerged during the past three decades. High-risk clonal complexes of various species of bacteria have spread in the healthcare setting across large swathes of continents. We have been witnessing a dramatic expansion of some global clones/lineages of methicillin-resistant *Staphylococcus aureus* (MRSA) (Nübel et al., 2011; Grundmann et al., 2014), extended-spectrum β-lactamase (ESBL)-producing *Klebsiella pneumoniae* (Damjanova et al., 2008; Holt et al., 2015), ESBL-producing *E. coli* (Pitout and DeVinney, 2017), *Clostridium difficile* (He et al., 2013), and vancomycin-resistant *Enterococcus faecium* (VRE) (Willems et al., 2005) among others.

The question arises what may account for the “attack and successful takeover” of the clones?

Though the determinants of clonal dynamics are certainly multiple one of them appears to be “more equal than others.” Recent reports investigating the fitness cost associated with resistance to fluoroquinolones in MRSA, ESBL-producing *K. pneumoniae*, ESBL-producing *E. coli* and *C. difficile* clearly showed that favorable fitness linked to high-level resistance to fluoroquinolones substantially contributed to the emergence of the major international clones/lineages of these pathogens (Reviewed by Fuzi, 2016).

While minor clone/lineage strains of MRSA, ESBL-producing *K. pneumoniae*, ESBL-producing *E. coli* and *C. difficile* suffer considerable fitness cost upon developing high-level resistance to fluoroquinolones the major clone isolates retain most of their vitality allowing to achieve dominance in facilities where fluoroquinolones are in extensive use (Horváth et al., 2012; Knight et al., 2012; Holden et al., 2013; Tóth et al., 2014; Johnson et al., 2015; Wasels et al., 2015). The abundant literature on the clonal dynamics of these pathogens remains in complete agreement with the proposed “fitness cost advantage” concept (Reviewed by Fuzi, 2016).

The next question concerns the mechanism: in what respect do the major clone strains of MRSA, ESBL-producing *K. pneumoniae*, ESBL-producing *E. coli* and *C. difficile* differ from minor clone isolates that permits the preservation of fitness even at high levels of resistance to fluoroquinolones?

## The dominance of the double-serine mutations

Researchers investigating the clonal dynamics of the above pathogens are in concordance that the crucial difference between the major and minor clone strains lies in the formers' ability (or the inability of the latter) to develop favorable but not detrimental genetic alterations mutations in the DNA gyrase and topoisomerase IV genes (Horváth et al., 2012; Holden et al., 2013; Tóth et al., 2014; Johnson et al., 2015; Wasels et al., 2015). This capacity seems to be a prerequisite: successful clones have to be able to evolve mutations which confer high-level resistance to fluoroquinolones without appreciably compromising fitness (Fuzi, 2016). Consequently, the recognition of how individual mutations in the quinolone resistance-determining regions (QRDRs) in the DNA gyrase (*gyrA, gyrB*) and topoisomerase IV (*parC/grlA, parE/grlB*) genes impact pathogens is of utmost importance.

The two most successful clones/lineages of hospital-associated (HA)-MRSA in Europe and the United States, the ST22 and the new lineage of ST8, both characteristically carry two identical mutations: *gyrA* Ser84Leu and *grlA* Ser80Phe (Table 1; Witte et al., 2001; Coelho et al., 2011; Holden et al., 2013; Alam et al., 2015), while minor clone/lineage strains—suffering considerable fitness cost upon developing high-level resistance to fluoroquinolones—were reported to harbor other mutations or additional genetic alterations compromising vitality (Horváth et al., 2012).

**Table 1:** Dominant serine/double-serine mutations in “fluoroquinolone-associated” STs/lineages of multi-drug resistant pathogens (see references in text).

**Pathogen**	ST/lineage^*^	**GyrA**	**ParC/GrlA**
MRSA	ST5, 8, 22, 239	Ser84Leu	Ser80Phe
*K. pneumoniae*	ST11, 15, 147, 258	Ser83Phe, Ser83Ile	Ser80Ile
*E. coli*	ST131 H30	Ser83Ile	Ser80Ile
*C. difficile*	ribotype 1, 2, 5, 14, 15, 20, 23, 27	Thr82Ile	^**^
*S. pneumoniae*	ST63, 81, 83, 156, 180, 191, 236, 260,11892	Ser81Phe, Ser81Tyr	Ser79Phe, Ser79Tyr
*E. faecium*	lineages related to CC17	Ser83Tyr, Ser83Arg	Ser80Ile, Ser80Arg ^***^
*P. aeruginosa*	to be established	Thr83Ile	Ser87Leu ^***^

* *The lists, with the exception of E. coli, are not complete*.

** *The species does not carry the parC gene*.

*** *Some of the strains are void of parC mutations*.

Interestingly these double-serine mutations were also observed in rapidly expanding local strains (sublineages) of other sequence types of MRSA (Takano et al., 2008; Lozano et al., 2012; Chakrakodi et al., 2014; Khokhlova et al., 2015). Moreover, the double-serine mutations were reported the most frequent fluoroquinolone resistance mechanism in MRSA in multiple studies investigating the prevalence and genetic background of antibiotic resistance without determining the strains' clonal affiliation (Takahata et al., 1996; Deplano et al., 1997; Kaatz and Seo, 1998; Sierra et al., 2002; Noguchi et al., 2005; Iihara et al., 2006; Coskun-Ari and Bosgelmez-Tinaz, 2008).

Though the double-serine mutations seem to remain a “trademark” of successful HA-MRSA strains/lineages in the adult healthcare setting where fluoroquinolones are used extensively the isolates may also harbor additional DNA gyrase and/or topoisomerase IV mutations (Sierra et al., 2002; Noguchi et al., 2005; Iihara et al., 2006; Takano et al., 2008; Chakrakodi et al., 2014).

In a similar fashion to MRSA the corresponding double-serine residues were observed to mutate also in the strains of the major international sequence types of ESBL-producing *K. pneumoniae* (*gyrA* Ser83Phe or Ser83Ile; *parC* Ser80Ile) though will often be complemented with additional QRDR mutations (Alouache et al., 2014; Tóth et al., 2014; Nagasaka et al., 2015; Park et al., 2015; Zhou et al., 2016; Sekyere and Amoako, 2017). In contrast, fluoroquinolone resistant minor ST strains—losing considerable fitness (Tóth et al., 2014)—were reported to be either void of DNA gyrase/topoisomerase IV mutations; carried a “non-serine” mutation or harbored just one of the favorable serine mutations (Tóth et al., 2014; Nagasaka et al., 2015; Sekyere and Amoako, 2017).

Moreover, all ST258 strains—which are genetically related to the major ST11 group isolates and are largely responsible for the global dissemination of carbapenemases—carry the *gyrA* Ser83Ile, *parC* Ser80Ile double mutations (Table 1; Bowers et al., 2015).

Strains of the sole international ST131 H30 lineage of ESBL-producing *E. coli* also carry the two equivalent double-serine mutations (*gyrA* Ser83Leu or sometimes Ser83Ile and *parC* Ser80Ile). Furthermore, they harbor usually three or sometimes two supplementary mutations; typically: *gyrA* Asp87Asn, *parC* Glu84Val, and *parE* Ile529Leu (Table 1; Paltansing et al., 2013; Johnson et al., 2015; Kim et al., 2016; Röderova et al., 2017).

In contrast to the above species *C. difficile* carries a threonine residue in the corresponding 82 *gyrA* position and, interestingly, its genome is void of topoisomerase IV (Dridi et al., 2002). The major international ribotypes/lineages of *C. difficile* characteristically carry the *gyrA* Thr82Ile mutation (Table 1; Spigaglia et al., 2010; Wasels et al., 2015). The *gyrA* Thr82Ile mutation can either be harbored as a single genetic alteration or combined with additional *gyrA* and/or *gyrB* mutations (Huang et al., 2009, 2010; Spigaglia et al., 2010; Walkty et al., 2010; Lin et al., 2011; Dong et al., 2013; Aogáin et al., 2015; Kuwata et al., 2015).

In summary, the command of the double-serine mutations in specific positions in the DNA gyrase and topoisomerase IV genes has been shown to be a dominant feature of multi-drug resistant major STs/lineages in at least three species: *S. aureus, K. pneumonia*, and *E. coli*. In addition the carriage of an equivalent *gyrA* mutation in *C. difficile* is typical for the international ribotypes/lineages of the pathogen. These observations argue for a salient role for fluoroquinolones in the selection of these STs/lineages. Moreover, they strongly suggest that apart from incurring resistance to fluoroquinolones these mutations should confer some additional favorable trait on the bacteria.

## The energy balance associated with individual QRDR mutations

In a fluoroquinolone environment bacteria will be able to extensively disseminate if they can achieve high-level resistance against these antibiotics without suffering appreciable fitness cost.

It is well-established that the binding of fluoroquinolones to the DNA gyrase and topoisomerase IV molecules is mediated primarily by four amino acids. Two serine residues: *gyrA* Ser83 and *parC* Ser80 (based on *E. coli* numbering) and an acidic residue four amino acids downstream of both respective positions (Aldred et al., 2014). Recently a secondary binding mode for fluoroquinolones was demonstrated involving downstream *gyrA* and *gyrB* sequences (Mustaev et al., 2014; Malik et al., 2016).

The evolvement of QRDR mutations mainly reflects the mechanism of fluoroquinolone binding: the majority of the mutations observed to date in various bacteria will mostly affect the serine and/or acidic residues mentioned above though mutations affecting additional residues especially in the QRDR region are also common (Aldred et al., 2014; Hooper and Jacoby, 2015).

In most cases a single mutation in only one of the two target enzymes confers ≤ 10-fold drug resistance and mutations in both enzymes are usually required to attain a 10 – 100-fold rise in MIC value (Reviewed by Aldred et al., 2014). Thus, strains in general need to evolve minimum two but often even more mutations to reach the expedient level of fluoroquinolone resistance.

Though mutations at the crucial serine residues—in contrast to acidic mutations—did not appear to adversely affect catalytic activity in the absence of fluoroquinolones (Aldred et al., 2014) a review of fitness studies performed with various species carrying diverse QRDR mutations presents a more nuanced picture.

The literature supporting the beneficial nature of the *gyrA* Ser83 mutations are abundant.

The Ser83Ala *gyrA* mutation in *E. coli* was initially demonstrated to have no impact on the catalytic activity of the enzyme (Barnard and Maxwell, 2001) and subsequently both the Ser83Ala and Ser83Leu mutations were shown by multiple authors to confer a slight fitness advantage on the isolates (Komp Lindgren et al., 2005; Marcusson et al., 2009; Machuca et al., 2015; Huseby et al., 2017).

Similarly the *gyrA* Ser83Phe mutation was reported to be associated with some fitness gain in both *Salmonella typhimurium* (Giraud et al., 2003) and *S. Typhi* (Baker et al., 2013). In S. Typhi the *gyrA* Ser83Tyr mutation also proved energetically favorable (Baker et al., 2013).

In *Streptococcus pneumoniae* both Gillespie et al. (2002) and Rozen et al. (2007) failed to observe loss of fitness in an isolate with the *gyrA* Ser81Phe mutation and—supporting the results—Pan et al. (2017) did not demonstrate any change in the activity of DNA gyrase (topoisomerase II) carrying the Ser81Phe *gyrA* mutation. However, Rozen et al. (2007) reported some fitness cost with the *gyrA* Ser81Tyr alteration.

Furthermore, mutations in the corresponding serine residues of the gyrA gene in MRSA and *K. pneumoniae*—though tested less meticulously—were reported to be associated with no fitness cost. Horváth et al., 2012) failed to observe any loss of vitality during the *in vitro* induction of fluoroquinolone resistance in an MRSA isolate upon the onset of the Ser84Ile *gyrA* mutation. Tóth et al. (2014) reported that a strain of *K. pneumoniae* harboring a Ser83Tyr *gyrA* mutation with an MIC to ciprofloxacin of 0,5 mg/L showed excellent vitality relative to other isolates.

In some species—*Pseudomonas aeruginosa, Burkholderia cepacia, C. difficile, Campylobacter jejuni*—the corresponding *gyrA* residue participating in the binding of fluoroquinolones are not serine but threonine. Moreover, some slow-growing bacteria—*C. difficile, C. jejuni* and the *mycobacteria*—are void of the topoisomerase IV enzyme (Dridi et al., 2002) and QRDR resistance will, thus, be confined to the DNA gyrase genes.

In *P. aeruginosa* Agnello et al. (2016) observed favorable fitness in strains with the corresponding single QRDR mutation—*gyrA* Thr83Ile—in which no other important fluoroquinolone resistance mechanisms could be detected. Moreover, the *gyrA* Thr83Ile mutation in *B. cepacia* and the corresponding Thr86Ile change in *C. jejuni* were observed to result in some fitness advantage (Pope et al., 2008; Han et al., 2012). In addition *C. jejuni* strains carrying the latter mutation were shown to outcompete wild type strains in an *in vivo* model (Luo et al., 2005). Moreover, the Thr86Ile mutation was reported to positively impact the molecule's activity concluding in an improvement in the enzyme's physiology (Han et al., 2012).

In *C. difficile* the *gyrA* mutation characteristic for the major international ribotypes/lineages—Thr82Ile—was observed to be associated with just a minimal cost of vitality (Wasels et al., 2015). Nevertheless, the Thr82Ile *gyrA* mutation in *C. difficile* is sometimes combined with additional *gyrA* alterations which the strains might need to attain adequate level of resistance (Huang et al., 2009; Spigaglia et al., 2010; Walkty et al., 2010; Dong et al., 2013; Kuwata et al., 2015).

In summary we can say that the mutation of the *gyrA* serine/threonine residue responsible for the binding of fluoroquinolones almost always exerts some positive effect on the isolate's physiology but is often insufficient to individually incur the adequate level of resistance to fluoroquinolones.

The energy balance of the second mutation of the double-serine pair - affecting the *parC* serine 80 residue in *E. coli*- proved more equivocal and was individually tested in just two species. In *E. coli* the *parC* Ser80Ile mutation's impact on fitness was investigated by Marcusson et al. (2009) who reported a slight fitness cost in the isolate. In contrast in *S. Typhi* Baker et al. (2013) observed a small fitness gain with the same mutation.

In *S. pneumoniae* one of the mutations in the corresponding *parC* residue (Ser79Phe) was observed to be associated with some fitness cost (Rozen et al., 2007), however, another mutation of the same residue (Ser79Tyr) did not result in any loss of vitality (Gillespie et al., 2002; Rozen et al., 2007).

The remaining QRDR mutations in both the DNA gyrase and topoisomerase IV genes were observed to mostly engender more or less fitness cost in various species (Komp Lindgren et al., 2005; Rozen et al., 2007; Pope et al., 2008; Marcusson et al., 2009; Baker et al., 2013; Wasels et al., 2015).

The exception is the *parC* Asp83Tyr mutation that was reported to confer some fitness advantage on *S. pneumoniae* (Rozen et al., 2007). Interestingly a Glu84Lys mutation in the corresponding *parC* residue in *S. aureus* will seriously compromise the enzyme's activity, however, if a proline substitution is located in the same position (Glu84Pro) the molecule's physiology remains intact (Hiasa, 2002).

## The energy balance associated with multiple QRDR mutations

Interestingly QRDR mutations will confer diverse MIC elevations for fluoroquinolones in various species.

Among bacteria thoroughly tested, the rise in MIC value for fluoroquinolones as a consequence of individual QRDR mutations proved low in *E. coli*, and in *S. Typhi* (Marcusson et al., 2009; Baker et al., 2013; Machuca et al., 2015). The elevation proved higher in *S. pneumoniae* (Gillespie et al., 2002; Rozen et al., 2007), *P. aeruginosa* (Agnello et al., 2016) and *B. cepacia* (Pope et al., 2008). The greatest rise in MIC was observed in *C. difficile* (Spigaglia et al., 2010; Lin et al., 2011; Wasels et al., 2015) and *Campylobacter* (Ge et al., 2005; Jesse et al., 2006).

Though we do not have comparable data for MRSA and *K. pneumoniae* it is well-established that both pathogens are capable of developing high-level resistance to fluoroquinolones by evolving double or triple QRDR mutations which suggests that an elevation in MIC value associated with individual mutations should be higher than in *E. coli*.

The highest MIC values for fluoroquinolones observed in *C. jejuni* and *C. difficile* may be related to the topoisomerase IV enzyme deficiency in these species (Cooper et al., 2002; Dridi et al., 2002; Ambur et al., 2009). These pathogens harbor just a single fluoroquinolone target molecule—DNA gyrase—whose mutation should result in a greater barrier for fluoroquinolones since a second enzyme is not available to bind to and compromise.

Since individual QRDR mutations elicit just a very slight increase in the level of fluoroquinolone resistance in *E. coli* (Marcusson et al., 2009; Machuca et al., 2015) the strains need to accumulate multiple genetic changes to collectively generate the adequate level of resistance. This is the reason why the sole international lineage of ESBL-producing *E. coli* (ST131 H30) characteristically carries four or rather five well-defined mutations (Paltansing et al., 2013; Johnson et al., 2015; Kim et al., 2016; Röderova et al., 2017).

The evolution of a really favorable array of mutations remains a serious challenge. Exclusively strains of the global ST131 H30 lineage proved capable to accomplish this feat and develop a suitable assortment of mutations that remains the principal determinant permitting the global dissemination of this group (Johnson et al., 2015; Fuzi, 2016). As already mentioned the double-serine mutations—affecting prominent binding spots for fluoroquinolones—form part of this genetic combination (Johnson et al., 2015).

Some excellent data are available on the fitness balance generated by combinations of three of the five QRDR mutations evolved by the ST131 H30 *E. coli* lineage. Marcusson et al. (2009) observed that the double-serine combination conferred significantly higher level of resistance to fluoroquinolones than other “pairs” of QRDR mutations, however, they were associated with a slight fitness cost. Interestingly when the double-serine mutations were completed with the *gyrA* Asp87Asn change the MIC of the isolate rose considerably and a very slight fitness benefit was observed.

Machuca et al. (2015) also observed that the double-serine mutations conferred the highest MIC value—relative to other pairs of mutations—for fluoroquinolones, nonetheless, they reported a significant fitness gain in their isolate and confirmed the favorable nature of the “triple arrangement” including an additional *gyrA* Asp87Asn.

Diverse clonal affiliation of the isolates in these studies could have influenced findings.

Furthermore, Huseby et al. (2017) very recently affirmed that the double-serine combination confers the highest MIC value for fluoroquinolones and demonstrated that the *parC* Ser80Ile mutation is acquiring an increasing selective advantage with an increasing fluoroquinolone concentration. This fitness benefit can not be detected in the absence of fluoroquinolones. They also showed that the triple mutation—*gyrA* Ser83Leu, Asp87Asn, and *parC* Ser80Ile—have a fitness advantage over other triple combinations in the presence of ciprofloxacin (Huseby et al., 2017).

The energetically positive balance generated by the addition of the individually detrimental acidic gyrase mutation to the double-serine changes and the observation that the fitness benefit of the double-serine mutations materialized only in the presence of fluoroquinolones are essential findings reflecting the nuanced effect of combined mutations.

Though the impact of the fourth and fifth *E. coli* mutations (*parC* E84V, *parE* I529L) have not been individually tested the global success of the ST131 H30 isolates carrying these genetic traits (Johnson et al., 2015) strongly suggest that they should complement the other mutations favorably: the dissemination of the clone has been demonstrated to be linked to the fitness benefit associated with its high-level resistance to fluoroquinolones (Johnson et al., 2015).

In *S. Typhi* the fitness balance associated with the double-serine mutations has not been investigated however a fitness gain was demonstrated for various other pairs of mutations (Baker et al., 2013). Nevertheless, the various double mutations tested conferred just a slight elevation of MIC for fluoroquinolones. Interestingly a fitness cost was reported for the *gyrA* Ser83Phe, Asp87Gly, *parC* Ser80Ile triple mutations—which almost correspond to the triple mutations investigated in *E. coli* (Baker et al., 2013). Consequently—in a similar fashion to *E. coli*—if fluoroquinolones will ever select a major multi-drug resistant *S. Typhi* clone or lineage it will certainly have to harbor more than three QRDR mutations.

In contrast to *E. coli* and *S. Typhi* the double-serine mutations (*gyrA* Ser81Tyr or Ser81Phe and *parC* Ser79Tyr or Ser79Phe) incur higher level resistance to fluoroquinolones in *S. pneumoniae* (Table 1; Pletz et al., 2004; Adam et al., 2007; Hsieh et al., 2010; Rodríguez-Avial et al., 2011; Schmitz et al., 2017).

Interestingly conflicting reports have been published on the fitness balance of the double-serine mutations in this species. While both Johnson et al. (2005) and Rozen et al. (2007) observed a fitness cost associated with the double-serine mutations of *gyrA* Ser81Phe and *parC* Ser79Tyr in genetically-engineered strains, Gillespie et al. (2002) found that the identical double-serine mutations failed to compromise vitality subsequent to *in vitro* induction. Unfortunately the clonal affiliation of the isolates - that could have influenced the findings - have not been determined in these studies.

Prior to the advent of vaccination fluoroquinolone resistant *S. pneumoniae* strains used to often disseminate clonally and the isolates primarily carried double-serine mutations (Pletz et al., 2004; Adam et al., 2007; Hsieh et al., 2010; Rodríguez-Avial et al., 2011). Interestingly strains from these fluoroquinolone-resistant *S. pneumoniae* clones did not show any fitness cost and even have sometimes exceeded wild type strains in propagation assays (Rudolf et al., 2011).

The anti-pneumococcal vaccination has reduced the rate of fluoroquinolone-resistance in *S. pneumoniae*. The previous resistant clones receded and have been partly replaced by novel STs. Some of these novel strains still proved capable of developing the double-serine mutations (Ceyssens et al., 2016; Schmitz et al., 2017). However, in a few locations “postvaccination strains” had not been able to evolve the energetically favorable double-serine changes. Interestingly these strains having failed to evolve the mutations conferring major elevations in MIC values often accumulated multiple alternative QRDR mutations to attain resistance to fluoroquinolones (Ceyssens et al., 2016; Takeuchi et al., 2017). The dearth of the energetically favorable double-serine mutations might have been responsible for their apparent inability to disseminate extensively (Ceyssens et al., 2016; Takeuchi et al., 2017).

The energy balance associated with the individual QRDR mutations have not been tested in MRSA, however, the dominance of these mutations in the successful healthcare associated STs and lineages—as mentioned earlier—argues strongly for a favorable fitness effect. In addition, Horváth et al., 2012) showed that minor clone strains of CA-MRSA suffering significant fitness costs upon developing resistance to fluoroquinolones either do not carry the double-serine mutations or harbor additional detrimental genetic changes. Moreover, Holden et al. (2013) demonstrated that the emergence of the major international ST22 clone in the United Kingdom has been linked to the evolvement of a novel clade wielding superior fitness relative to earlier variants and harboring—among other genetic features—the double-serine mutations.

In the major international STs of ESBL-producing *K. pneumoniae* the double-serine mutations (*gyrA* Ser83Ile, *parC* Ser80Ile) conferred high-level resistance to an isolate and were observed to be associated with just a very small fitness cost (Tóth et al., 2014). The triple-mutations (*gyrA* Ser83Phe, Asp87Ala, and *parC* Ser80Ile) also incurred high-level resistance and were associated with some loss of vitality in a strain-specific fashion (Tóth et al., 2014). In addition, as mentioned above, the preeminence of the double-serine mutations in the international STs of ESBL-producing *K. pneumoniae* also supports *the* beneficial nature of this combination.

In *P. aeruginosa* carrying a threonine residue in the *gyrA* 83 position Kugelberg et al. (2005) observed that the double *gyrA* Thr83Ile and *parC* Ser80Leu mutations conferred high-level resistance to fluoroquinolones but were associated with fitness cost. It has to be noted that the genetic change reported initially as *parC* Ser80Leu is actually *parC* Ser87Leu since the serine residue is located in the latter position in *P. aeruginosa* (Lee et al., 2005).

Recently Agnello et al. (2016) reported that the *parC* Ser87Leu mutation introduced into strains of P. aeruginosa already carrying the *gyrA* Ser83Ile mutation was associated with a slight fitness cost in five of six isolates. However, it conferred a small fitness benefit on one of the isolates suggesting that clonal affiliation is relevant also in this species. In addition, authors observed that the fitness cost was influenced by the genotype of the type III secretion system harbored by the isolates (Agnello et al., 2016). Moreover, the dominance of the *gyrA* Thr83Ile alteration often associated with the *parC* Ser87Leu change in fluoroquinolone resistant strains of *P. aeruginosa* (Higgins et al., 2003; Lee et al., 2005; Agnello and Wong-Beringer, 2012; Yang et al., 2015; Araujo et al., 2016; Nouri et al., 2016) argues strongly for the salient role of the double-threonine/serine mutations in selecting groups of bacteria with favorable fitness also in this species.

The influence genetic (clonal) differences exert on the evolution of favorable QRDR mutations in *P. aeruginosa* was reflected in the findings of Jørgensen et al., 2013). These investigators induced resistance to fluoroquinolones *in vitro* in their *P. aeruginosa* isolates. Surprisingly none of the strains proved capable of developing the *gyrA* 83 and *parC* 87 mutations which—as mentioned above—are the most common genetic alterations in clinical isolates. In contrast all of them evolved QRDR mutations in unorthodox positions (Jørgensen et al., 2013).

Further studies could unequivocally establish whether or not the double-threonine/serine mutations contributed to the clonal dynamics in *P. aeruginosa*.

In *B. cepacia* the *gyrA* Thr83Ile and *parC* Ser80Leu double mutations were reported to have conferred high level resistance to fluoroquinolones but were associated with significant fitness cost.

Species which lack the topoisomerase IV enzyme—*C. difficile* and *C. jejuni*—can-not evolve genetic combinations equivalent to the double mutations mentioned above. In the major international clones of *C. difficile* a single QRDR mutation—*gyrA* Thr82Ile—proved optimal, concurrently ensuring proper fluoroquinolone MIC value and adequate fitness balance (Table 1; (Wasels et al., 2015)). Some major clone strains may also carry a second QRDR mutation, that may be a consequence of excessive fluoroquinolone exposure, or more rarely a distinct mutation in the *gyrA* or *gyrB* genes (Spigaglia et al., 2010).

Similarly to *C. difficile* a single mutation—*gyrA* Thr86Ile—may confer the adequate level of resistance to *C. jejuni* (Iovine, 2013). It will often be supplemented with an additional QRDR alteration (Iovine, 2013).

In contrast to MRSA, ESBL-producing *K. pneumoniae*, ESBL-producing *E. coli* and *C. difficile* in *C. jejuni* the ability to evolve favorable mutation(s) was not restricted to a few STs/lineages. To our understanding *C. jejuni* may be the single pathogen among bacteria reviewed in this paper that has efficiently adapted to resistance against fluoroquinolones “as a species.” In this bacterium a multitude of genetic variants - not just a few clones—have been able to develope favorable mutational resistance (Luo et al., 2005; Zeitouni and Kempf, 2011). This excellent capacity of adaptation is most certainly the consequence of the hyperplasticity of the *C. jejuni* genome (Stahl and Stintzi, 2011).

In summary we can say that in a number of species which harbor both the DNA gyrase and topoisomerase IV genes the success of individual clones/lineages depend on their ability to evolve the double-serine—or equivalent—mutations often supplemented with additional appropriate changes in the QRDR regions.

It is well-established that *E. coli* belongs to this group. There is also *in vitro* and epidemiological evidence that the expansion of the major STs/lineages of both ESBL-producing *K. pneumoniae* and HA-MRSA have been the consequence of a similar process (see above). Moreover, there is some circumstantial evidence that a similar mechanism may have contributed to the dissemination of fluoroquinolone resistance in *S. pneumoniae* and *P. aeruginosa* (see above).

Slow-growing bacteria like *C. difficile* and *C. jejuni* lacking the topoisomerase IV gene had to embrace different strategies. However, even in these species the ability to evolve an energetically favorable *gyrA* threonine mutation proved crucial for dissemination—for major clones (*C. difficile*) or for the whole species (*C. jejuni*)—in a fluoroquinolone environment.

## What drives evolution?

As we have seen individual STs/lineages of various species differ in their ability to develop favorable QRDR mutations. This trait, however, has been shown to be amenable to change. Clones/lineages are capable of undergoing a remarkable metamorphosis and improve their ability of evolving QRDR mutations.

ST239 and ST8 MRSAs remain minor clones in Hungary and the latter was observed to lack the favorable double-serine genetic combination (Horváth et al., 2012). In addition they are also on the retreat in many parts of Europe (Grundmann et al., 2014). In contrast the new lineage of the ST8 MRSA in the United States and some ST239 strains in Asia have been reported to carry the double-serine mutations that should have contributed to the success of these pathogens in those areas (Takano et al., 2008; Chakrakodi et al., 2014; Alam et al., 2015; Khokhlova et al., 2015).

Similarly, while some novel “postvaccination clone” isolates of *S. pneumoniae* investigated in Belgian and Japanese studies failed to develop the favorable double-serine mutations (Ceyssens et al., 2016; Takeuchi et al., 2017), other “novel clone-strains” in Belgium and Germany succeeded in evolving the genetic combination (Ceyssens et al., 2016; Schmitz et al., 2017).

Clones/lineages of additional species are certainly not impervious to a similar metamorphosis.

Consequently minor clone/lineage isolates can sometimes “assume” the ability to evolve favorably. It remains of utmost importance to identify the determinants driving this evolutionary process.

## The evolutionary background of the double-serine residues

The double-serine residues confer susceptibility to an important group of antibiotics, fluoroquinolones. In addition, the replacement of the gyrase serine residue (residue 83 in *E. coli*) is associated with a slight fitness advantage. The question arises, if these amino acids are not favorable why have they been evolutionarily preserved across species?

The most probable answer is that they confer resistance to naturally occurring substances microbes are sometimes exposed to.

Japanese scientists demonstrated a couple of years ago (Hiramatsu et al., 2012) that fluoroquinolone resistant *S. aureus* strains carrying the double-serine mutations (in the *gyrA* 84 and *parC* 80 residues) were all susceptible to a natural antibiotic (nybomycin, a quinolone-dione) produced by several streptomyces species. Conversely fluoroquinolone susceptible wild type isolates harboring both serine residues proved resistant. The *parC* serine mutation proved pivotal in incurring resistance (Hiramatsu et al., 2012). Interestingly nybomycin also showed activity against fluoroquinolone resistant *enterococci* (Hiramatsu et al., 2012).

In addition fluoroquinolone resistant *S. aureus* isolates carrying the double-serine mutations have also shown susceptibility to a natural flavonoid substance—apigenin—produced by various plants while fluoroquinolone susceptible isolates remained resistant (Morimoto et al., 2015). In contrast to nybomycin here the mutation of the *gyrA* 84 residue played an essential role in conferring protection (Morimoto et al., 2015).

It is tempting to speculate that ancient bacteria extensively exposed to herbal flavonoids (Hiramatsu et al., 2012) in order to protect themselves had to compromise the activity of their gyrase enzymes and consequently their fitness by evolving the *gyrA* serine residue. Thus, the change of the *gyrA* serine residue will prove favorable-in a non-flavonoid environment.

## The significance of additional fluoroquinolone resistance mechanisms and virulence factors

Though the QRDR mutations are pivotal in conferring high-level resistance to fluoroquinolones some additional mechanisms also form relevant part of the bacterial defense hardware.

Various efflux systems are well-established to participate in resistance against fluoroquinolone type antibiotics (Aldred et al., 2014). However, in contrast to some QRDR mutations the energy balance of efflux—unless it incurs just a slight elevation in the MIC value for fluoroquinolones—remains negative. An abundance of papers investigating diverse species of bacteria reported fitness cost associated with the activity of various efflux systems (Sánchez et al., 2002; Komp Lindgren et al., 2005; Marcusson et al., 2009; Martinez et al., 2009; Tóth et al., 2014; Machuca et al., 2015; Huseby et al., 2017). Consequently, bacteria are usually not supposed to extensively deploy their energy-consuming “efflux defense” if they are capable of evolving the energetically more favorable *gyrA* serine mutation combined with the *parC* serine change which together - and sometimes with additional mutations - will raise the MIC value for fluoroquinolones to the “requisite” level. And this is really what has been observed in those species which have already been investigated in that respect.

Tóth et al. (2014) tested the efflux activity in ESBL-producing major and minor ST *K. pneumoniae* isolates. Fluoroquinolone resistant major ST strains—harboring either the double-serine mutations or the double-serine plus one triple combination—were observed to have similar propagation rates to susceptible isolates and failed to show any efflux activity. Conversely efflux activity could be demonstrated in three of the four minor ST isolates tested. The fluoroquinolone resistant minor ST strains were void of QRDR mutations, carried a single mutation other than the double-serine or harbored just one of the two serine mutations (Tóth et al., 2014). The efflux systems of the wild type minor ST strains—prior to *in vitro* induction with ciprofloxacin - were inactive (Tóth et al., 2014). It seems obvious that these isolates proved unable to evolve the favorable QRDR mutations and could not, thus, raise their MIC values to the adequate level. Then trying just to survive the exposure to the increasing concentrations of ciprofloxacin turned on their efflux systems. However, driving the efflux required energy which resulted in fitness cost slowing the growth rates of the isolates conferring a serious liability vis-à-vis “well-energized” major ST strains in a fluoroquinolone environment.

Similarly Johnson et al. (2015) reported that in multi-drug resistant *E. coli* minor ST strains carried fewer QRDR mutations than the ST131 H30 major lineage isolates but showed significantly greater efflux activity. Comparable studies in other species have not been performed. Nevertheless, the use of efflux for the removal of fluoroquinolones from the bacterial cell should be common in minor ST isolates in many bacteria that may account for some findings reported in the literature. Bagel et al. (1999) reported just a minimal fitness cost associated with the *gyrA* Ser83Leu mutation in a strain of *E. coli* in which the alteration was genetically engineered. In contrast they observed a large fitness cost in a clinical isolate carrying just the same single mutation. It is tempting to suggest that the unorthodox finding was due to the clinical strain being a minor clone isolate. It carried a single QRDR mutation suggesting that it must had been exposed to fluoroquinolones. Since the sole mutation should not have conferred the adequate MIC value it could have turned on the energy consuming efflux.

In *P. aeruginosa* Kugelberg et al. (2005) observed high fitness cost in a clinical isolate with high MIC value carrying a single *gyrA* Thr83Ile mutation, however, an active efflux was also detected. Moreover, Agnello et al. (2016) demonstrated dissimilar fitness in clinical isolates harboring the sole Thr83Ile mutation and showed that all the strains with substantial loss of vitality run a highly active efflux system.

Additional fluoroquinolone resistance mechanisms include *qnr* proteins protecting target enzymes from quinolones and a variant of the aminoglycoside acetyltransferase enzyme - *aac(6-)-Ib-cr*- conferring reduced susceptibility to ciprofloxacin by *N*-acetylation of its piperazinyl amine. Both mechanisms confer low-level resistance to fluoroquinolones (Aldred et al., 2014).

*Qnr* proteins were reported to have been associated with both some fitness benefit and a slight fitness cost in *E. coli* (Michon et al., 2011; Machuca et al., 2014). They are, interestingly, sometimes absent from the ST131 major clone strains of ESBL-producing *E. coli* (Aoike et al., 2013; Paltansing et al., 2013; Reyna-Flores et al., 2013; Guillard et al., 2014). The evolutionary edge associated with the *qnr* proteins remains obscure. They have been observed to confer protection against some quinolone-like compounds of herbal origin but, interestingly, not against quinolone type quorum sensing molecules produced by pseudomonas species (Kwak et al., 2013).

In contrast to *qnr* proteins the carriage of the *aac(6-)-Ib-cr* gene is common in both major ST/lineage ESBL-producing *K. pneumoniae* and ESBL-producing *E. coli* isolates (Musumeci et al., 2012; Reyna-Flores et al., 2013; Guillard et al., 2014; Tóth et al., 2014; Park et al., 2015; Zhou et al., 2016).

Apart from diverse fitness costs associated with resistance to fluoroquinolones fitness balance linked to other groups of antibiotics have not been seriously implicated in the selection and promotion of the international STs/lineages of the above pathogens. Virulence factors, however, have often been suggested to prime the advancement of various major groups of bacteria.

A review of all the major virulence factors of the above pathogens remains way beyond the scope of this paper. Thus, only salient issues related to virulence in the international STs/lineages of ESBL-producing *E. coli*, ESBL-producing *K. pneumoniae* and HA-MRSA will briefly be covered.

Virulence factors have not been seriously implicated in the expansion of the *E. coli* ST131 H30 lineage. It is well-established that the lineage is a diverse group with regard to virulence. It consists of multiple “virotypes” showing distinct degrees of pathogenicity (Blanco et al., 2011; Olesen et al., 2014; Miyoshi-Akiyama et al., 2016). Moreover, non-ST131 *E. coli* strains were reported to be just as virulent as some ST131 isolates (Ciesielczuk et al., 2015). Though, Cha et al. (2016) recently alleged that the ST131 clone was more virulent than others, Han et al. (2017) showed that ST131 strains fail to excel in aggregate virulence. Furthermore, Dahbi et al. (2014) reported that ST131 isolates show diverse virulence and low-virulence isolates are common among them.

In ESBL-producing *K. pneumoniae* biofilm formation was supposed to further the expansion of the major ST11 sequence type (Andrade et al., 2014; Melegh et al., 2015), however, Diago-Navarro et al. (2014) observed “heterogeneity” in the formation of biofilm in ST258 strains of *K. pneumoniae*—a close relative of the ST11 major sequence type. Moreover, Kong et al. (2012) questioned the role of biofilm formation in the development of systemic infection with *K. pneumoniae*. Recently a possible role for yersiniabactin was proposed in ST15 *K. pneumoniae* isolates (Zhou et al., 2016). However, Lee et al. (2016) showed that yersiniabactin production is common also in minor clone isolates. Moreover, the probably most virulent *K. pneumoniae* sequence type, ST23 does not belong to the major groups of the pathogen (Struve et al., 2015).

In MRSA the production of biofilm and the carriage of the arginine catabolic mobile element (ACME) have been mentioned as potentially essential clonal enhancers, however, the incidence of these virulence factors remains at variance with the observed clonal shifts in HA-MRSA. The issue was recently reviewed (Fuzi, 2016).

In summary, virulence factors seem to play only a secondary role compared with diverse fitness cost associated with fluoroquinolone resistance in the selection and promotion of international ST/lineage isolates in the multi-drug resistant variants of at least three species.

## The possible role of the double-serine QRDR mutations in the emergence of the major international lineages of VRE

As we have shown data available on *S. pneumoniae* and *P. aeruginosa* hint at a possible role for fluoroquinolones in the dissemination of these pathogens. However, in an additional species—vancomycin-resistant *E. faecium* (VRE)—both the genetic and epidemiological evidence for the involvement of a similar process remains much stronger.

It is well-established that a few lineages related to a single international clone of VRE (CC17) dominate the hospital setting worldwide (Willems et al., 2005; Leavis et al., 2006a; Cattoir and Leclercq, 2013; Freitas et al., 2016; Guzman Prieto et al., 2016) and the literature suggest that it also was selected and promoted by the extensive use of fluoroquinolones.

The process of clonal reduction was spectacular and has been well-documented in VRE. The use of a glycopeptide antibiotic in animal husbandry—avoparcin—promoted the dissemination and enteric carriage of the pathogen in the European population in the 1990s. An abundance of papers reported the often widespread carriage of VRE across the continent (Bingen et al., 1991; Jordens et al., 1994; Gordts et al., 1995; Moulin et al., 1996; van den Bogaard et al., 1997). These isolates of animal origin, however, proved unable to disseminate in the adult hospital setting (van den Braak et al., 1998).

Interestingly, not only the VRE clones of animal origin failed to disseminate in hospitals. The multiple clones of human VRE observed in that period (Clark et al., 1993; Sadowy et al., 2013) also suffered a substantial reduction and subsequently several lineages all related to a single clone of the pathogen (CC17) emerged in adult hospital wards worldwide (Willems et al., 2005; Cattoir and Leclercq, 2013). These internationally dominant groups of pathogens differ significantly from other human VRE clones and lineages and even more from those VRE clones adapted to various animals species (Willems et al., 2000, 2005; Werner et al., 2008; Qin et al., 2012).

Though these bacteria are called VRE, interestingly, some strains from these lineages remain susceptible to vancomycin (Willems et al., 2005; Leavis et al., 2006a; Coombs et al., 2014). However, most of them seem to be resistant to fluoroquinolones (Leavis et al., 2006a; Werner et al., 2010).

Moreover, Cattoir and Leclercq (2013) showed that the CC17 clone emerged as an ampicillin and fluoroquinolone resistant group that only subsequently acquired resistance to glycopeptides. Furthermore, the observation that among the various VRE clones/lineages exclusively the CC17-related strains harbor the well-known double-serine fluoroquinolone resistance mutations in the DNA gyrase and topoisomerase IV genes (Leavis et al., 2006b; Sadowy et al., 2013) constitutes a strong circumstantial evidence for the selective and promoting role of fluoroquinolones in the dissemination of these bacteria.

The CC17-related *E. faecium* strains' exceptional capacity to evolve the DNA gyrase and topoisomerase IV mutations was recently shown by French scientists. de Lastours et al. (2017) demonstrated that non-CC17-related lineages of VRE are generally unable to develop the favorable *gyrA* and *parC* mutations. 48 healthy volunteers were treated with ciprofloxacin and then, the fluoroquinolone resistance in *E. faecium* strains isolated from the enteric commensal flora was investigated. Remarkably none of the commensal *E. faecium* strains proved capable of evolving the favorable *gyrA* and *parC* mutations.

Though resistance to ampicillin has been a feature of CC17-related lineages for a long time (Cattoir and Leclercq, 2013) it is highly unlikely that it could have played a major role in the promotion of these groups of pathogens. Antibiotic resistance surveillence data collected by the European Union clearly show that not only vancomycin-resistant *E. faecium* strains are resistant to ampicillin; the overall resistance rate in most countries remains over 75 percent (http://atlas.ecdc.europa.eu/public/index.aspx).

Virulence factors have also been implicated in the advancement of the CC17-related lineages. Primarily the *esp* gene involved in biofilm formation was supposed to make a crucial contribution (Top et al., 2008). There is no doubt that the esp gene has contributed to the extensive dissemination of these lineages. However, the available data from the literature strongly suggest that its role remains less significant than the ability to evolve favorable QRDR mutations.

While it is well-established that the rate of fluoroquinolone resistance is consistently high in CC17-related VRE lineages the carriage rate for *esp* varies strongly across studies (Klare et al., 2005; Khan et al., 2008; Galloway-Peña et al., 2009; Mato et al., 2009; López et al., 2010; Bjørkeng et al., 2011; Fallico et al., 2011; Kang et al., 2014).

## The impact of fluoroquinolone use on the incidence of “major STs/lineages” of multi-drug resistant pathogens

The available information on the influence of diverse fitness cost associated with fluoroquinolone resistance on the clonal dynamics of various multi-drug resistant pathogens suggests that the use of fluoroquinolone type antibiotics should profoundly impact the incidence of these bacteria provided they belong to “major STs/lineages.”

Since most HA-MRSA strains in adult hospital wards belong to “major STs” worldwide a decrease or increase in fluoroquinolone consumption should have a prompt effect on the pathogen's incidence. This is really what has been observed. A plethora of papers almost concordantly demonstrated that there is a direct link between the use of fluoroquinolones and the incidence of HA-MRSA in adult departments. The related literature was recently reviewed (Fuzi, 2016).

The proportion of the major ribotypes of *C. difficile* proved also high in the European surveillance system (Fawley et al., 2016). The United Kingdom launched a campaign about 10 years ago to reduce the incidence of *C. difficile* infections by decreasing the use of fluoroquinolones. The results were spectacular: a dramatic decline in the number of reported cases was instantly observed which was associated with the partial replacement of the major ribotypes of the pathogen by minor clones (Reviewed recently by Fuzi, 2016). Moreover, an abundance of papers from a variety of countries show that a reduction in the consumption of fluoroquinolones will result in a decrease in the incidence of *C. difficile* infections (Reviewed recently by Fuzi, 2016).

Though there are no reliable data on the prevalence of “major ST/lineage” strains of the remaining multi-drug resistant pathogens mentioned above it is certain that their proportion is smaller than those of HA-MRSA and *C. difficile*. Thus, we can expect a significant impact on the incidence of these bacteria—subsequent a decrease in the use of fluoroquinolones—exclusively if we have established that the proportion of the “major ST/lineage” isolates is high in the studied facility.

Consequently the judicious use of fluoroquinolones should be dynamic and a function of the clonal composition of the local multi-drug resistant pathogens. Should the proportion of the “fluoroquinolone-associated STs/lineages” exceed a “threshold” the consumption of fluoroquinolones should be abandoned. Conversely, if the proportion of the “fluoroquinolone-associated STs/lineages” remains low fluoroquinolones can certainly be used to great advantage.

Further studies exploring clonal affiliations would be needed to elucidate the impact of fluoroquinolone use on the incidence of various multi-drug resistant pathogens. These studies would also help to establish the “threshold proportions” for “major STs/lineages” indicators for the suspension or resumption of fluoroquinolone therapy. Though the judicious use of fluoroquinolones would not permanently resolve the problem of antibiotic resistance it would reduce the proportion of “major ST/lineage” strains and lower the incidence of multi-drug resistant pathogens ameliorating the situation.

In summary the ability of some STs/lineages of various multi-drug resistant bacteria to evolve the favorable DNA gyrase and topoisomerase IV mutations—primarily in the double-serine residues—advanced their dissemination in fluoroquinolone environments. The reason(s) why these “competent” pathogens are more adept to favorably mutate than most strains of the same species and how previously “non-mutating” strains develop the capacity to mutate remain to be elucidated. Furthermore, our better understanding of the mechanism of clonal dynamics permitting a more judicious use of fluoroquinolone type antibiotics would allow for the amelioration of the general antibiotic resistance situation.

## Author contributions

MF devised and wrote the manuscript. DS and RC helped with their advice and provided relevant literature.

### Conflict of interest statement

The authors declare that the research was conducted in the absence of any commercial or financial relationships that could be construed as a potential conflict of interest.
